# A comparison study between CT angiography with 64-multislice spiral computed tomography and selective X-ray coronary angiography

**DOI:** 10.3892/etm.2013.905

**Published:** 2013-01-17

**Authors:** LUYANG CHENG, SHENHONG JING, YINA ZHANG

**Affiliations:** 1 Department of Geriatrics, The Second Affiliated Hospital of Harbin Medical University, Harbin Medical University, Harbin, Heilongjiang 150086, P.R. China; 2 Department of Cardiology, The Second Affiliated Hospital of Harbin Medical University, Harbin Medical University, Harbin, Heilongjiang 150086, P.R. China

**Keywords:** computerized tomography angiography, 64-slice spiral computed tomography, X-ray, coronary angiography

## Abstract

The aim of this study was to evaluate the diagnostic value of 64-multislice spiral computed tomography (64-MSCT) for coronary stenosis compared with selective X-ray coronary angiography (SCA). Patients with chest pain, chest tightness or coronary stenosis received SCA and they acted as the controls. The sensitivity and accuracy of 64-MSCT were analyzed as compared with SCA. Images from 64-MSCT were obtained for 95 patients. For the diagnosis of myocardial bridge, 64-MSCT coronary CT angiography (CTA) is superior to SCA. In cases of mild coronary stenosis, combined with clinical symptoms, patients may choose to receive conservative treatment instead of SCA. However, cases of moderate coronary stenosis should receive SCA to determine the diagnosis. In conclusion, no difference was observed between 64-MSCT coronary CTA and SCA in their ability to exclude true negative diagnoses and diagnosing true positives of coronary disease. The 64-MSCT coronary CTA method produces improved image quality for diagnosis of coronary stenosis and is a non-invasive, reliable and effective method for the diagnosis of the severity of coronary stenosis.

## Introduction

Coronary heart disease (CHD) is one of the most common diseases and has a serious impact on human health and safety (1). It has become one of the top causes of mortality in China. The prevalence of CHD is increasing and sudden mortality is often due to this sudden disease. Detecting CHD as early as possible before clinical symptoms appear is important. Selective X-ray coronary angiography (SCA) is one of the standard tests for CHD; however,it has several disadvantages, including high-cost, invasive diagnosis and the performance of the procedure necessitates considerable time and skill of highly trained physicians. In the past few years, non-invasive diagnosis of CHD has made great progress. With the improvement of computerized tomography (CT) hardware and software technology, 64-multislice spiral CT (64-MSCT) CT angiography (CTA) has become a common, non-invasive diagnostic method. In this study, we assessed the clinical application of 64-MSCT CTA compared with SCA, based on previous randomized studies (2,3).

## Patients and methods

### Patient characteristics

A total of 95 patients with suspected obstructive coronary artery disease received 64-MSCT. Of these, 67 patients were identified to have coronary stenosis. There were 43 males and 24 females, aged 41–81 years (average age, 65 years) with regular sinus rhythm. For patients with a fast heart rate, the rhythm could be controlled below 70 bpm, without Betaloc allergy effects. The study was conducted in accordance with the Helsinki Declaration II and was approved by the Institutional Review Board of Harbin Medical University. All patients provided written informed consent which was signed by the patient or their families.

### Methods

The heart cover area was set at 120 mm with a fast rotation speed of 0.35 sec per rotation. The mean pitch of the heart scan was 0.23. The detector width was 64×0.625 mm and the time of the heart scan was 4.57 sec. The blood circulation time was evaluated by a test bolus. The injection rate of the contrast media was 5 ml/sec. The range of the scan covered the whole heart. The patient was instructed to maintain an inspiratory breath hold. The whole process was performed by an interventional cardiologist.

## Results

**Figure 1.**
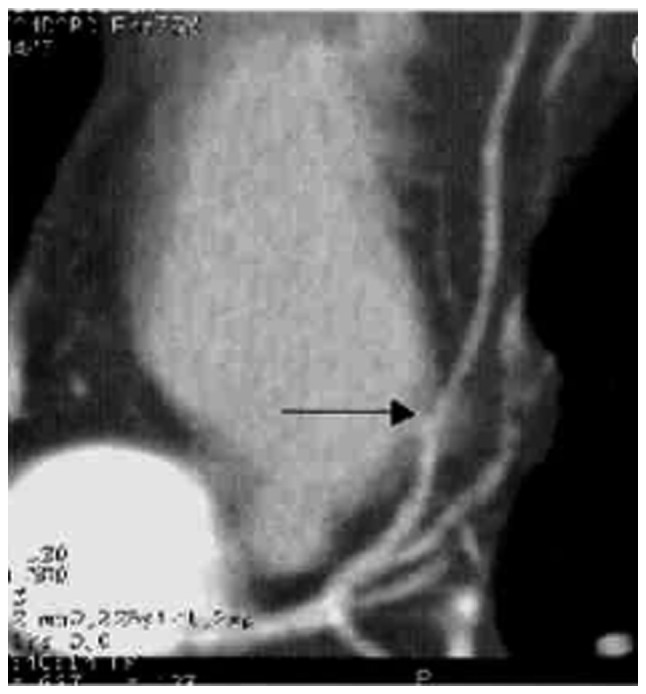
A myocardial bridge was observed in the proximal segment of the left anterior descending coronary artery by computerized tomography angiography.

**Figure 2.**
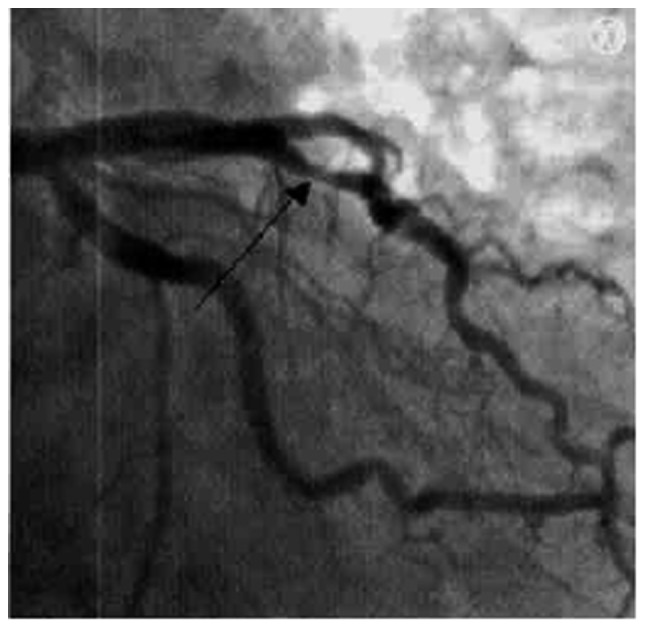
A myocardial bridge was observed in the proximal segment of the left anterior descending coronary artery by selective X-ray coronary angiography.

**Figure 3.**
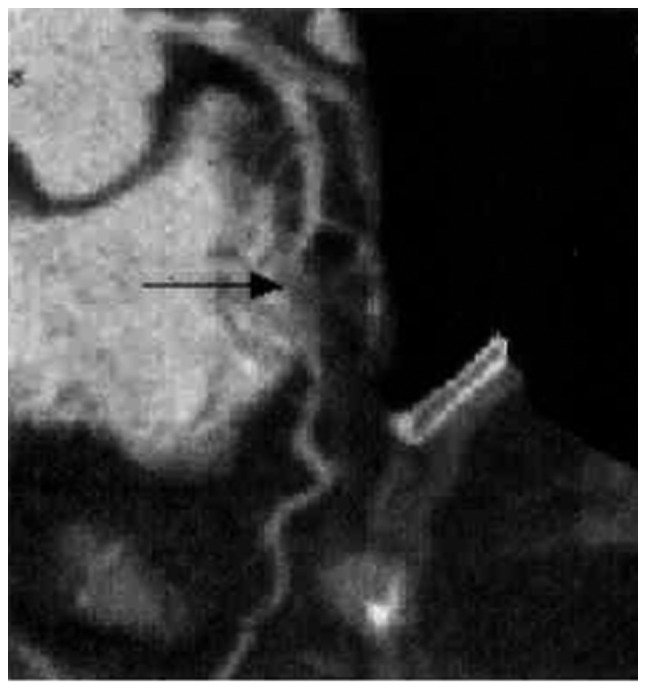
Computerized tomography angiography demonstrated that the middle of the left anterior descending coronary artery dips into and underneath the heart muscle.

**Figure 4.**
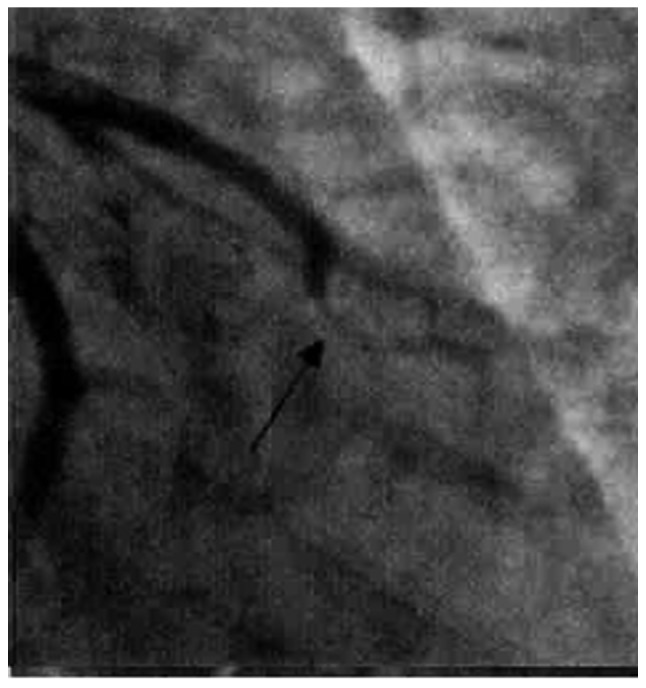
Coronary angiography demonstrated that there was severe stenosis near the occlusion in the middle of the left anterior descending coronary artery.

All 95 patients received 64-MSCT. All the main blood vessels and major branches were smooth and there was no stenosis in 18 patients. Coronary myocardial bridge (CMB) was detected in 10 patients. Among the 67 patients with coronary stenosis, we detected mild coronary stenosis (<50%) in 19 patients, moderate coronary stenosis (50–70%) in 26 patients and severe coronary stenosis (>75%) in 22 patients. Of the 18 patients without coronary stenosis 11 presented symptoms, including chest distress and chest pain. The SCA results revealed no coronary stenosis. In addition, 4 of the 5 CMB patients were diagnosed with SCA (Figs. 1 and 2) and 1 patient was diagnosed with severe coronary stenosis (Figs. 3 and 4).

**Figure 5.**
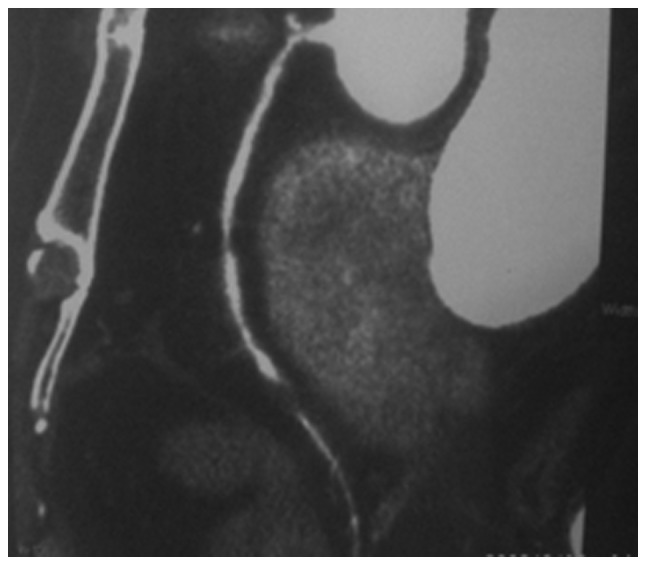
Stenosis was detected in the right coronary artery by computerized tomography angiography. One is moderate stenosis in the proximal area and the other is severe stenosis in the distal area.

**Figure 6.**
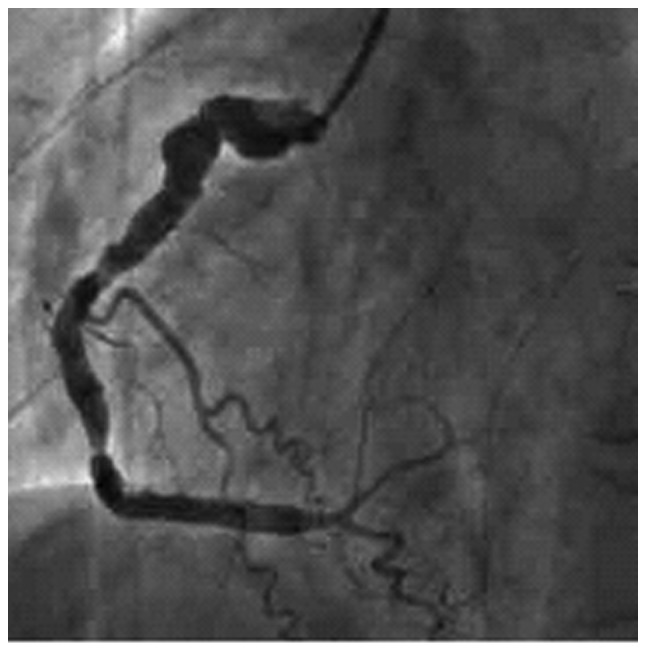
Stenosis was detected in the right coronary artery by selective X-ray coronary angiography. One is moderate stenosis in the proximal area and the other is severe stenosis in the distal area.

Among the patients with coronary stenosis, the SCA results of 5 patients with mild coronary stenosis revealed that the level of coronary stenosis was ∼30, 60, 30, 30 and 30%, respectively. In 9 of the 26 patients with moderate coronary stenosis the SCA results were consistent with the 64-MSCT results. However, 17 patients had severe coronary stenosis and percutaneous transluminal coronary intervention treatment confirmed the main and major branch of the diseased blood vessel for these patients with severe coronary stenosis (Figs. 5 and 6).

## Discussion

CHD is one of the main factors threatening human health and it is also one of the main causes of mortality in China. Diagnosing coronary stenosis as early as possible and initiating treatment helps to prevent disease progression. The most common clinical methods for coronary stenosis are 64-MSCT CTA and SCA, and SCA is more accurate at this stage. SCA is not only used for diagnosis, but also for intervention therapy. However, the conduit method has certain risks due to the invasive property, particularly for patients who are not suitable for intervention therapy. Additionally, SCA often increases the financial burden and certain risks. Therefore, the reliable noninvasive 64-MSCT CTA has attracted attention. In the past few years, the 64-MSCT hardware has improved, the scanning time has reduced dramatically and the time and spatial resolution have increased rapidly. In addition, the breath artifact is avoided when the heart rate is <70 bpm and the heart motion artifact is avoided. It also provides an improved image quality, which clearly shows the coronary artery. Therefore, 64-MSCT CTA is a common and popular technique (4).

In 64 patients with CHD, 64-MSCT CTA was performed and the results on coronary stenosis reveal no difference when compared with SCA diagnosis. Therefore, 64-MSCT CTA may be used clinically to identify a true negative value when the symptoms of CHD do not agree with the electrocardiogram (ECG) diagnosis (5). A CMB is a band of heart muscle that lies on top of a coronary artery, instead of underneath it. With a CMB, part of a coronary artery dips into and underneath the heart muscle and then comes back out again (6). The band of muscle that lies on top of the coronary artery is called a bridge. With research on anatomy, pathology and blood hemodynamics, it was identified that a CMB may cause myocardium ischemia under certain conditions. The diagnosis and therapy of CMB is becoming increasingly important. SCA is a classic method for diagnosis, which shows a milking-like effect, where the CMB is squeezed by contraction of surrounding muscle and the constriction disappears during diastole. The 64-MSCT CTA method displays the coronary artery and surrounding myocardial area; therefore, it has a better diagnostic result than SCA on CMB. Furthermore, 64-MSCT CTA avoids a false positive diagnosis. CMB is often misdiagnosed as lumen stenosis and stent implantation is applied; however, this may cause the lumen to break. Therefore, 64-MSCT CTA has become a better option for diagnosing CMB (7,8).

In this controlled study, we compared 64-MSCT CTA and SCA diagnostic results and identified that SCA is capable of diagnosing a mild or moderate coronary stenosis and 64-MSCT CTA detects a mild coronary stenosis. We suggest that when 64-MSCT CTA detects a mild coronary stenosis with typical symptoms and the ECG shows no clear evolvement, a conservative therapy should be performed and the patient’s condition strictly monitored during the therapy. Then, SCA should only be performed when necessary.

In this study, when comparing moderate coronary stenosis detected by 64-MSCT CTA with SCA diagnosis for the same patients, SCA revealed that more than half of the patients had severe coronary stenosis and required coronary stent therapy. Therefore, we suggest that SCA is performed when a moderate coronary stenosis is detected by 64-MSCT CTA, to prevent delay in treatment and possible heart infarction. For severe coronary stenosis, detected by 64-MSCT CTA, we observed a clear agreement between 64-MSCT CTA and SCA diagnosis (9). In summary we suggest that 64-MSCT CTA is used at the early stages for those with no CHD symptoms, mild symptoms or no significant variation in ECG results. It is not necessary to perform a check with SCA if nothing shows up from the 64-MSCT CTA diagnosis. For those patients who have clear symptoms, significant variation in ECG results and 64-MSCT CTA diagnosis reveals moderate to severe coronary stenosis, a further SCA check is necessary. A stent implantation therapy should be performed when the coronary stenosis level reaches ≥70% (10).

Further development is required for 64-MSCT CTA to improve the time resolution and spatial resolution. This enables an advanced CT detector to reach an equal quality with SCA (0.2×0.2×0.2mm) (11). With the development of tube rotation speed and re-build technology, the non-invasive CTA is considered the next generation method of SCA.
